# Continuous Traumatic Stress: Examining the Experiences and Support Needs of Women After Separation From an Abusive Partner

**DOI:** 10.1177/08862605221132776

**Published:** 2022-11-13

**Authors:** Joanne Hulley, Khai Wager, Tim Gomersall, Louis Bailey, Gill Kirkman, Graham Gibbs, Adele D. Jones

**Affiliations:** 1 University of Central Lancashire, UK; 2 University of Oxford, UK; 3 University of Huddersfield, UK; 4 University of Salford, UK

**Keywords:** intimate partner violence, domestic abuse, battered women, intervention/treatment, PTSD, CTS

## Abstract

Intimate partner violence causes significant, long-lasting harm to almost one-third (27%) of the world’s population of women. Even when women leave abusive relationships, some men continue to exercise control over their ex-partners through psychological control, threats, violence, stalking, and other forms of harassment. In this qualitative study, 52 purposively sampled women who self-identified as victims or survivors of intimate partner violence (IPV) from male partners were interviewed. Data were analyzed with a theoretically informed thematic analysis, supported by Nvivo® software. We found that leaving a violent relationship was a long-term process fraught with difficulty and ongoing risks of psychological harm. The concept of Continuous Traumatic Stress (CTS), first developed to understand the impact of state-sponsored violence and war, was found to be a particularly useful tool for the analysis of the impact of post-separation abuse. Additionally, CTS encourages researchers and practitioners to think anew about resilience-centered approaches to improving protection and access to justice for female victims.

This study examines the impact of post-separation abuse on women who have experienced intimate partner violence (IPV) using the idea of continuous traumatic stress (CTS) as a conceptual aid. Our data come from interviews with 52 UK-based heterosexual women who self-identified as “victims” or “survivors” of IPV. However, for the purpose of this article we use the term “survivor.” The CTS construct was used for the analysis of the impact of post-separation IPV, by applying five key domains of the CTS construct, as outlined by Eagle and Kaminer (2013) to organize our analysis. The CTS construct was developed to capture the experiences of people living in contexts of *ongoing* violence in apartheid South Africa, where posttraumatic stress disorder (PTSD) diagnoses and interventions were insufficient in addressing the emotional and psychological impacts of ongoing state violence. Expanding its application, this study investigates whether the CTS construct is useful in capturing women’s continuing experiences of post-separation IPV.

IPV is the most prevalent form of violence against women globally, with the WHO’s 2018 global estimates, based on ever-married/partnered women aged 15 years and older, placing lifetime prevalence at 26%. Notably, IPV starts early, with almost one-quarter of ever-married/partnered adolescent girls aged 15 to 19 years having experienced violence from an intimate partner at least once (World Health Organisation [WHO], 2021). In the United Kingdom, lifetime prevalence of IPV is 24%, broadly in line with global prevalence (WHO, 2021).

In the United Kingdom, the Domestic Abuse Act 2021 provides a definition of ‘domestic abuse’ as abusive behavior between personally connected individuals of 16 years of age or older, where “abusive behavior” can refer to “physical or sexual abuse,” “violent or threatening behavior,” “controlling or coercive behavior,” “economic abuse,” or “psychological, emotional or other abuse.” Importantly, domestic abuse is broader than IPV as it includes as “personally connected individuals” relatives that may not be, or have ever been, intimate partners.

We use the phrase “IPV” rather than domestic violence to indicate the continuation of abuse beyond the domestic sphere and we also use “abuse,” rather than violence, to signify non-physical forms of control. While we acknowledge that some women continued to feel trapped and victimized by perpetrators, we stress the importance of recognizing women’s resilience, as indicated by our use of the term “survivor.” We also use both “perpetrator of abuse” and for ease of expression, its shorthand “perpetrator.”

## Post-separation Abuse and Control

Women remain at high risk of lethal and nonlethal violence from abusive ex-partners (Brownridge, 2006), with one Canadian study reporting that separated and divorced women experience nine and four times, respectively, the prevalence of violence from their ex-partners than do married women (Brownridge et al., 2008). There are limited UK-based studies on the prevalence of post-separation abuse, though in one UK study 90% of women reported experiencing post-separation abuse (Sharp-Jeffs et al., 2018), and in a study based in Wales, looking at experiences of help-seeking at the juncture of leaving an abusive relationship, 10 out of 12 women reported experiencing post-separation abuse (Wydall & Zerk, 2020). Furthermore, 18% of UK femicide cases included in the 2020 Femicide Report, that were by an intimate partner, occurred post-separation.

The Domestic Abuse Act, Section 3, (2021) recognizes that children are victims of domestic abuse and are no longer considered just “witnesses.” Perpetrators use children in various ways to harm, harass, threaten, and control their partners (Mbilinyi et al., 2007), especially post-separation when potential avenues of control are reduced (Bancroft et al., 2012; Hayes, 2012). In cases where a woman has obtained a restraining order, abusers can use children as a continued line of unwanted contact (Bancroft et al., 2012). Abusers may also threaten to take children away, to harm them, physically abuse them or, in cases where the mother has primary custody, delay returning them (Hardesty & Ganong, 2006). They may also turn children against their mothers by blaming them for negative events (Hayes, 2012). In one study, in the United States, of 156 women who had recently experienced IPV, 88% of women reported that their ex-partners had used their children against them (Beeble et al., 2007). Rivera et al. (2012) found most women experienced secondary victimization through the mediation process used for child custody negotiations and some experienced re-victimization by their abuser during the process.

Emotional abuse is also prevalent post-separation (Zeoli et al., 2013). Emotional abuse reported in one study included stalking/controlling behaviors such as sending threatening text messages, making harassing telephone calls, harassing women at work, sitting outside ex-partners’ houses, and vandalising property (Zeoli et al., 2013). Post-separation, Hayes (2012) suggests that physically abusive men may switch from physical abuse to more covert forms of abuse, particularly if courts become involved in their separation. In some cases, perpetrators are able to present a charming persona in court (Jaffe et al., 2008) or to friends and family of ex-spouses (Hayes, 2012) in order to discredit reports of abuse.

Ending an abusive relationship often leaves women financially depleted and this presents opportunities for their abusive ex-partners to continue their abuse. When examining financial abuse within IPV, Stylianou (2018) highlights three forms of such abuse: economic control, employment sabotage, and economic exploitation. Economic control relates to limiting access to resources, monitoring the use of money, hiding jointly earned money, preventing an intimate partner from having their own bank account, and lying about shared resources (e.g., Sanders, 2015). Economic control may extend to controlling child support payments, for example, to exert control (Hardesty et al., 2008). Economic sabotage involves abusers preventing victims from obtaining or maintaining employment. This can include forbidding, discouraging, or interfering with employment, for example, women who were interviewed in one study reported being forced to leave their jobs after receiving abuse at work from their ex-partners (Humphreys & Thiara, 2003). Economic sabotage may also include preventing victims from sleeping the night before an interview, destroying work clothes, disabling the car, and failing to keep childcare promises (Tolman & Raphael, 2000). Finally, economic exploitation refers to behavior which intentionally aims to deplete financial resources (Postmus et al., 2012). This may be achieved through stealing money, borrowing money in the victim’s name, refusing to pay bills or mortgage payments, and stealing or damaging possessions. Researchers have suggested that abusive ex-spouses may use litigation as a form of coercive control, which in addition to representing emotional abuse functions as economic abuse, exacting unmanageable financial costs (Jaffe et al., 2003).

## Continuous Traumatic Stress

All these forms of abuse may continue after separation and give rise to considerable trauma that is not confined to the past, the pre-separation period. Consequently, this article employs the construct of CTS (Eagle & Kaminer, 2013; Straker & the Sanctuaries Counseling Team, 1987) to organize our analysis of the experiences of ongoing control and abuse by ex-partners against women who have left violent relationships. CTS provides a means of assessing the impact of living in contexts of *ongoing* violence and was first proposed by mental health professionals working with victims of torture and violence during the excesses of state oppression in apartheid South Africa (Straker & the Sanctuaries Counseling Team, 1987). Straker and her team developed the concept of CTS after noting that PTSD diagnoses and interventions failed to capture the experiences and needs of victims of violence in contexts in which the prospect of ongoing victimization was likely and when living conditions and the failure of laws and discriminatory provision of protective services increased its likelihood.

Before applying the CTS construct to the experiences of post-separation IPV, it is important to first locate the construct in relation to PTSD and also, to consider the relationship between IPV and definitions of trauma. CTS diverges from PTSD in four respects: CTS emphasizes the importance of context, the temporal location of the stressor conditions, the complexity of discriminating between real and perceived or imagined threat, and the absence of external protective systems. The CTS model thus represents a shift away from a preoccupation with the symbolic and psychosocial signifiers of past incidents toward a future-oriented approach. To enable an individual to regain a sense of control, a CTS intervention strategy focuses on coping strategies for managing *ongoing* threats where there is a strong possibility of further victimization. Instead of energy spent on the deconstruction and processing of arousal events, a CTS strategy proposes the conscious construction of internal strategies for ensuring safety for the future and minimizing the impact of ongoing traumatic stress.

## IPV, Trauma, and PTSD

Trauma can be defined as an individual’s psychosocial stress response to violence. IPV, like other forms of violence, can trigger trauma, but importantly, it is rarely a single event and more commonly reflects ongoing abusive behaviors and actions. Traumatized people may experience hyperarousal, avoidance, and intrusive symbols of past violence even as they live with present violence, all of which can also be symptoms of PTSD. Explained as a chronic emotional response to prior trauma (American Psychiatric Association [APA], 2005), PTSD can lead to panic attacks and nightmares, depression, suicidal ideation, and drug and alcohol misuse, all of which may also arise because of IPV. Despite these connections, the initial research which established PTSD as a psychiatric diagnosis excluded women’s experiences of domestic violence (van der Kolk et al., 2007). Consequently, the inability of IPV survivors to feel safe, and the impact of IPV and other forms of gender-based violence (including child abuse) on self-esteem and self-concept have led to alternative diagnoses, including, an over-emphasis on psychosis and neurosis (specifically, borderline personality disorder) in assessing women’s mental health. Feminist critics suggest this reflects a pathologizing of the ways that women respond to gendered abuse and oppression (Shaw & Proctor, 2005). While it is important to highlight the narrow foundation on which traumatology theory has been built, this is not therefore, a call for the integration of IPV trauma symptoms into the PTSD classifications. Indeed, survivors of IPV often present with symptoms that extend beyond the narrow range of difficulties that can be identified through a PTSD lens alone (Courtois & Gold, 2009). The trauma response to violence, whether in lived reality, perceived, or anticipated does not necessarily indicate that the event is in the past or is finite. PTSD is therefore an inadequate basis for understanding the impact of trauma stress response to whole life or continuous experiences of IPV. This article makes a case for the consideration of the CTS construct in filling this gap.

### Method

This article draws on data collected in 2019 as part of a qualitative study on IPV in the UK carried out by the authors (the full dataset is held under embargo due to the sensitive nature of the data at the University of Huddersfield: https://doi.org/10.34696/x1hq-d175).The study utilized a cross-sectional qualitative design—data collected at one time-point through guided reflective interviews with perpetrators and survivors of IPV. This paper uses only the data from the interviews with women survivors in heterosexual relationships. The objectives of the study were derived from a preliminary literature review and sought to explore what the survivors of IPV understood it to be, the types of violence they experienced, the role of technology and social media, their childhood experiences, disclosure, their experiences of IPV, and its impact on both themselves and their families.

The study used convenience, purposive sampling techniques to identify potential participants. Participants were recruited primarily through professional networks, key stakeholder groups, and snowballing. Fifty-one women who self-identified as victims or survivors of IPV participated in interviews which lasted an average of 1.5 hours. Interviews were digitally recorded and transcribed. Initially a thematic analytic approach was used based on a set of *a priori* themes derived from previous work undertaken by some of the team and from the preliminary literature review. The NVivo software program (v12) (QSR International, Boston, MA) supported coding and data management. During coding, the team had regular meetings to assess the accuracy of the coding and the appropriateness of the themes. To ensure content validity and to minimize researcher bias, a sample of coding was cross-checked between researchers and agreement reached on coding selection.

Ambiguities about the application of the themes were resolved and, in some cases, led in an inductive fashion to new key themes. The *a priori* themes focused on the dimensions of IPV, the experience of abuse, the effects on the survivor and their family and friends, help seeking (barriers and behaviors), strengths and resilience, and the types of support needed. But other issues emerged that had to do with the longer-term experience of IPV and especially the experience of survivors after they had separated from their abusers. New themes and coding that reflected this were added to the coding scheme. One issue that emerged strongly from the long-term experience of IPV and from the experience of abuse was the continuous trauma experienced by the women. Therefore, initial findings were subject to further thematic analysis. The underpinning theory used for this second stage of analysis was the Continuous Traumatic Stress (CTS) construct. Eagle and Kaminer (2013) developed this construct to explain the implications of living with ongoing risk of violence victimization and stress-related trauma. Though originally derived from peace and conflict studies, in using this framework as an interpretative tool in the examination of IPV survivors’ experiences, the authors seek to deepen understandings of the long-term impact of domestic abuse. Five key domains were included in the interpretative framework, reflecting key concerns embedded in CTS research:

1. The ongoing temporality of traumatic stress
2. The complexity of discriminating between real and perceived or imagined threat
3. Anticipatory anxiety, loss of control, and its impact
4. Strengths and resilience—alertness to risks, adaptivity
5. The absence of external protective systems

Ethical approval was granted by the University of Huddersfield. The participants’ identities were anonymized and post-interview counseling was made available in the event that participants should experience re-traumatization. Participants were provided with an information sheet detailing the nature of the study and informed that they could withdraw at any stage of the process. Data collection and data management were subject to stringent ethical protocols to safeguard women’s privacy rights and all transcripts and digital recordings were secured through password protection.

### Findings

Table 1 describes the participant characteristics—all participant names and identifying details have been changed to protect participant anonymity. Fifty-two women aged from 17 to 63 years participated; two women were divorced, eight were married, one was separated, and 39 were single at the time of the interviews (this data for two women is missing). Nineteen women were in employment and descriptions of socio-economic status spanned a range from “struggling to get by” to “well-off” with the majority of women (*n* = 27) describing their financial situation as “earning enough to meet family needs.” Thirty of the women had children and seven had three or more children. The majority of the women were white British and only eight women were from black and minority ethnic backgrounds. Additionally, all our participants identified as heterosexual and experienced IPV at the hands of male partners. The absence of LGBTQ+ voices from our study, as well as the small number of black women, and women from ethnic minority backgrounds, are important caveats to bear in mind when interpreting our findings.

**Table 1.** Participant Demographics.

Age (Mean, SD)	Number of Children (*n*, %)	Economic Circumstances (*n*, %)	Marital Status (*n*, %)
28 ± 9	None: 15 (29%)	Well off: 5 (9.8%)	Single: 39 (76.5%)
	One: 14 (27%)	Earn enough to meet family needs: 26 (51%)	Married: 8, (15.7%)
	Two: 8 (17.6%)	Struggle to get by: 14 (27.5%)	Separated: 1, (1.9%)
	Three or more: 7 (13.7%)	Data missing: 7 (11.8%)	Divorced: 2 (3.8%) No data: 2 (1.9%)
	Data missing: 7 (11.7%)		
	Pregnant: 1 (1.9%)		

### The Temporal Location of the Stressor Conditions

One of the key findings of this research was the sequential and progressive stressor conditions women faced. Mirroring other research on CTS, where violence and trauma forms part of the overall life trajectory, it was rarely the case that women had experienced just one violent relationship which continued to affect them post-separation. Instead, violence for our participants was chronic, for some, spanning back to childhood and for most, continuing after separation from abusive partners. Past violence had never been a single, isolated event but a series of violent actions within the relationship which had escalated. For a large minority of women, this was the continuation of a history of childhood abuse. Sixteen participants had witnessed parental IPV or had directly experienced parental neglect, physical or sexual abuse as children. This led to low self-worth and the internalization of norms and beliefs about male violence which distorted perceptions about abuse and left women vulnerable to predation.

“Because I’d had the experience of my dad, I just thought that was normal in a relationship. . .” (Zoe).

Jasmine had experienced multiple forms of abuse by her parents and saw relationship abuse as simply the continuation of this:

“. . .physically, emotionally, mentally, anything you can really find to be honest. So, for a lot of my life it was just normal.”

Participants who experienced childhood abuse typically did not recognize the situations they were in as abusive and this contributed to violence acceptance:

“. . .part of being a victim of domestic violence erm, it became normal, so when the acts were carried out on me physically, it was just normal. . . and I accepted that for a long time.” (Eliza).

Nicky had been sexually abused by her stepfather from aged 4 to 12 years and at 16, her stepfather’s brother tried to rape her. When later, she disclosed the abuse to her husband, he reacted by blaming her and subjecting her to humiliation and shame. Nicky, like other women, was dealing with the effects of unresolved trauma which made her vulnerable to subsequent relationship abuse. Jane, who was sexually abused by a family friend for 4 years from the age of seven also illustrates this:

“It’s scarred my life, it never leaves. . .when things happen over and over again, it is like the norm but it does hurt and then it leaves people damaged. It’s left me damaged.”

Among the women with long histories of abuse, there were two perspectives on their entry into abusive relationships: some indicated that the normalization of abuse combined with low self-esteem impacted their relationship choices but others believed that abusive men had detected their vulnerabilities:

“. . . it’s like they’ve got a radar. . .they act like they’re not clever but they’re very clever. . .to pick up on that you’re a vulnerable person” (Karen).

“You see men actually see. . .they know that I must be vulnerable or I accept it. . .Basically I was put in this world just to get hurt. . .” (Jane).

Scarlett had faced sexual, physical and emotional abuse all of her life, to the extent that she believed she must have been to blame; she had no expectation that post-separation, other relationships would be any different. This finding helps to explain why several women had been in more than one abusive relationship. All of Eleanor’s relationships spanning 30 years, had been abusive and Karen had been in relationships with three violent partners. At least 11 of the participants had suffered more than one abusive relationship.

The stress and fear women lived with during the abusive relationship often continued post-separation. Women described ongoing harassment and threats after leaving their partners; this wore them down, threatened their jobs and livelihoods and created subliminal anxieties that sometimes dominated their lives. Louise was harassed from prison, where her ex-partner was serving a sentence for the abuse he had inflicted. He called each morning, lunchtime and evening to check where she was and wrote letters daily; the anticipatory anxiety this generated eroded any sense of safety. Elizabeth was stalked by her ex-partner and found the experience frightening and emotionally exhausting:

“. . .there was about 6 months where he was still following. . .About a year later. . .he found me at . . .and he messaged me to say “I’m outside your flat.” (Elizabeth).

Survivors sometimes experienced emotional pressure from ex-partners who threatened to self-harm or commit suicide if they did not return. Sienna faced this threat continuously for some time after separation, causing her severe mental stress:

“It was mental torture. . .. . .. . . I ended up taking an overdose because of him.” (Sienna).

For women who were mothers, ex-partners often used the children as a means of continuing coercive control. Having exposed children to violence and in some cases, causing them direct harm, men now posed as model fathers to gain custody or access to their children. A number of women had been forced to take legal action to limit the risks this posed to them and their children; proceedings which were often lengthy, arduous, and costly.

## Anticipatory Anxiety, Loss of Control, and Their Impact

Women described a high degree of anticipatory anxiety especially when contemplating whether to leave the relationship. They were acutely aware of their partner’s capacity for violence and the anticipation of retributive violence was derived from their assessment of the likelihood of future abuse. This assessment included past experiences but also, both explicit threats *and* implicit signifiers of harm intention, conveyed through a “look” or tone of voice.

Managing the risk of repercussive violence required different strategies depending upon the woman’s circumstances and in most cases meant either abandoning the idea of getting help or being exceedingly careful in doing so. When Victoria was injured by her partner, she “knew” that she should not seek medical help even though she needed emergency treatment and when Daisy decided to call the police about the escalation of danger in her relationship, she used another person’s phone. Managing anticipatory anxiety called for the conscious regulation of circumstance, including self-regulation. Elizabeth recalled her belief that she would be killed and she constantly reminded herself to keep silent about the abuse.

The women felt a degree of control over the situation while the abuse remained a private matter. This was not borne out, as violence often escalated anyway, but it was important for women to be able to control the stress of contemplating what might happen should they tell anyone about the abuse. Liz recalled the time when a neighbor contacted the police to report the sounds of violence coming from the house. As she opened the door to the police officer who responded to the call, the perpetrator stood behind her with a knife; terrified, she managed to mask her fear and sustain the façade that everything was under control. Evidence of women taking agentic control in managing the potential for future violence and the ongoing trauma this might cause was illustrated by Clare who stopped her mother from calling the police, fearing that if they did not arrive quickly, the intervening period would give her partner time to wreak revenge. Louise too, convinced her abuser would target her family, minimized the abuse she faced, even when it was recognized by others and refused to involve the police because she did not believe they would be protected.

## The Complexity of Discriminating Between Real and Perceived or Imagined Threat

Discerning whether the threat of future violence was real, perceived, or imagined was complicated by psychological abuse, and the erosion of self-belief. Where partners had criminal records, as was the case for several women, they felt more certain in their assessment of the risk of ongoing violence, although most did not learn of this until after they had either left the relationship or reported the violence. Haley was alarmed to discover that the perpetrator of her abuse was already known to the police because of previous violence.

“He’s been known to the police for a good ten years and his record is very disturbing. . . he has pending convictions that hasn’t even been sent to court yet. But his background is very, very horrifying. . .”.

Having previously been incarcerated, Victoria’s ex-partner said that if he was sent back to prison as a consequence of her reporting him, he would kill her when he got out; she considered this a very real threat. Mandy had been separated from her ex-partner for 4 months at the time of the interview and he continued to intimidate her:

“He’s told em [Mandy’s parents] he’s gonna stab me to bits when he comes home, so. . . When he comes home I hope he gets arrested anyway because he’s not been arrested for it. Something’s gotta happen about these threats, you know, it’s threats to kill.”

The violence Mandy had faced during her relationship of 6 years was extremely severe and often involved being stabbed and cut by knives. She had lived with the belief that he would change, now they were separated, she was absolutely sure he would not. Many women acknowledged that if they had been targeted then quite probably other woman would be too. Lisa’s ex-partner had raped a 14-year-old girl after they had separated and had he not been imprisoned, she was convinced she would have suffered retaliatory violence from him.

While it was not always the case that relationship violence portended post-separation abuse, some women felt able to intuit this, even if direct threats had not been issued. Claudia was so concerned about the safety of her and her son that she sought confirmatory evidence of her ex-partner’s previous history of domestic violence (the UK Domestic Violence Disclosure Scheme gives anyone the right to ask the police to reveal information indicating someone may be a risk for committing abuse). Although there was no factual evidence of risk, the police assigned a ‘critical mark’ to Claudia’s property (this is a UK crime prevention technique in which police place a marker on a survivor’s address so that they can respond more quickly to domestic violence incidents); she regarded this as a validation of her assessment of risk:

“. . .there was nothing disclosed from his past but they put a critical mark on my property. . .so it was when it was just me and my little boy in our place and I was thinking “what the hell?”

Some women had been so impacted by traumatic stress that this impaired their judgement. Liz’s partner had been accused of raping another woman and she recalled acting as a witness in his defense:

“I actually remember. . .sitting in front of the judge and testifying how wonderful he was. . .and that there’s no way he could rape somebody. . . And I sat there saying these things whilst he. . . was essentially raping me. . .looking back I feel horrified and. . .. ashamed of what I was doing, because I was defending a criminal, essentially.”.

A majority of women had experienced coercive control for so long that compliant behaviors were a part of their internal coping mechanisms and were self-sustained even when there was no actual threat of ongoing violence. This was described by Britney as “mental torture”: “. . . it scars you. Not outside of your body, it scars you inside. . . it scars you for life.” Blue exemplifies this emotional scarring. At the insistence of her husband, she had worn ankle bracelets with bells for 30 years and in this way, he had been able to control and monitor her movements. When he began an affair with her friend, inadvertently, it had been Blue who enabled him to continue undetected.

“Oh those are my bells because my husband made me wear them so he could hear me wherever I went. . .”

Even after she was divorced, Blue still felt as if she was under the control of her ex-partner and continued to wear the bells; “ I can’t go without them anymore. . .it’s ’cos my husband wanted to know where I was.”

## Strengths and Resilience—Alertness to Risks and Adaptation

There were many ways in which women demonstrated strength and resilience, both in surviving violent relationships and in being able to leave. Britney had been physically and emotionally abused for six years and lived with her two young children and partner in enforced poverty and isolation. She believed that she had been stripped of all her personal resources and as she had been rendered entirely dependent on her partner, never thought she would be able to escape. Despite this, she found a reservoir of strength and one day, when her partner was out of the house, she took up a bag of belongings and together with her children, took a train until she had put a hundred miles between them. She described it like this:

“You get up and pull your power and move on. You can get there. Just take that path. *You* can take that path. No-one else can take that path for you. . .I’m not saying it’s gonna be easy. It will be hard, and I’ve learned that. But it gets easier, and easier and easier. Day by day, if you just get through one day, the first other day you’re getting there.”

Eva had been forced to drop out of university by her partner but resumed studies post-separation; this gave her renewed focus. Similar strategies were seen throughout the dataset, with women picking up on thwarted ambition and finding jobs or taking courses, sometimes selecting subjects to help them understand their experiences, such as criminology and psychology. Women described feeling the need to regain control of their lives and immersing themselves in new environments helped them to adapt to their new circumstances. However, adaptation was a long and challenging process. Eva suffered from depression and was diagnosed with PTSD. She had self-harmed during the abuse as a means of coping and had often felt suicidal; she recalled her ex-partner’s response when she shared her feelings of desperation with him:

“I remember he called me one day and he said ‘you know what?’ Like you probably should just kill yourself.”

Eva was adapting to a life without her partner but had lost all her friends because of his violence and although she had the support of her mother, she was isolated and aware that she was at high risk of self-harming again as a means of dealing with the recurring nightmares and memories. Women who were mothers found great strength in their children. Chloe said that keeping her daughters safe had helped her to stay resilient during her abusive relationship and for Tess, it was thinking about her children’s needs that helped her to leave:

“. . .I’m living and my kids need me to live for them. . .I’m going to get up and I’m going to put my big girl. . . knickers on, I’m going to brush my hair back and I’m going to get on with it. . .”.

Being unable to access counseling, Rivers created her own internal strategy of what she called a “self-mothering, self-parenting technique, where you sort of create that nurturing voice in your head and it doesn’t always have to be that self-critic that’s the biggest voice.”

## The Absence of External Protective Systems

Within the UK, there does exist an extensive network of arrangements within comprehensive policy and legislative frameworks that seek, in principle at least, to safeguard women against IPV. These include local partnership agreements between the police and housing, health, domestic abuse agencies, refuges, and social services; dedicated support organizations, websites, helplines, and community, faith-based and women’s groups. However, there remain major challenges for survivors of IPV in accessing protection and justice and particular problems for minoritized and marginalized women (e.g., women from black and minority ethnic communities, disabled women, asylum seekers). The evidence suggests that, women typically experience 50 violent events before being able to get effective help (SafeLives, 2015) and the critical issue seems to lie in the resourcing of services, weak implementation and monitoring, and the failure to adequately center women’s rights and voices in policy development. For example, if survivors had a share in the property they lived in, regardless of how poor they were, they were prevented from obtaining legal aid. At the time of this study, women faced the prospect of having to sell their homes and making their children homeless to pay for legal representation or, having to cross-examine their abusers in court themselves. Although this ruling was overturned in January 2021 (Independent, 2021), many other institutional blockages to accessing help remain.

In the current study, multiple failures of the protective services were identified and neglect or indifference by professionals or organizations was noted by some women. Bystander apathy was also a contributing factor to the general lack of concern that women faced, as Rivers described:

“Any adult who would have had any part in stopping this from happening was not there. No one wanted to take any responsibility for me—parents, public who saw me being beaten up but did nothing.”

There were several disturbing accounts of the police failing to follow up with reports of violence and, of victim-blaming. Jasmine struggled to get the police to take her allegations of rape seriously. When she heard that three of her friends had also been hurt by the same man, she decided to get her own evidence, yet still no action was taken:

“So then that was obviously, me bringing it back up again. . . and I had like evidence on my phone, him messaging me saying I’m really sorry I raped you, I shouldn’t have done and they still didn’t do anything about it. But there was three of us and like I had like knife marks all over my body because he like literally cut me and all of it was well why did you go to his house?”

Rivers was attacked on a bus and reported the incident to the police. In this instance, the violence was dismissed because the perpetrator was disabled:

“. . .the police did nothing and it sort of came down to, he had a disability and they said that he didn’t know what he was doing, and then they literally didn’t write a report.”

In Eva’s case, the police *did* take her seriously but in attempting to convince her to prosecute the perpetrator, they failed to recognzse the absolute terror this instilled in her and their methods simply confirmed in her mind that she was responsible for the abuse. Here she describes the interview with a policeman:

“. . .you know ‘you really need to wake up,’ like he was quite forceful and he was saying like ‘you cannot carry this on. You need to do this, you need to do that. If you don’t do this, then this is gonna happen and you’re putting other girls’ lives in danger because you won’t prosecute him and what if he does this to someone else?’ And ‘you’re responsible for this guy’s actions’ and I was like, I just broke down in tears, and I thought ‘I don’t wanna be responsible for his actions anymore.’”

For some women, the response of agencies compounded their trauma. This was particularly the case concerning social services. Women described being treated as if *they* were responsible for their children’s exposure to violence and they were subject to a high level of scrutiny concerning their parenting. Post-separation, perpetrators threatened to fabricate child abuse and to report mothers to social services. Women believed that social service intervention would result in their children being taken away from them and this sometimes forced them to collude with the perpetrator’s account of violence. Adele’s child was hospitalized by her violent partner, and being unfamiliar with her legal position, she felt she had no choice but to go along with his explanation. While health care services were generally valued by women, health professionals rarely probed or enabled women to disclose and there were numerous accounts of “missed opportunities” for help-giving. For example, Eliza described being on a waiting list of 6 to 18 months’ duration despite suicidal feelings post- separation, while Lily described the reaction of health professionals to her experiences of abuse as believing it was “just teenagers being teenagers and girls being hysterical.”

## Discussion

In this article, we have examined women’s accounts of post-separation abusive relationships using the concept of CTS to inform the analysis. Our findings show that CTS is a helpful concept for informing theory and practice, and for supporting survivors of IPV throughout the separation and post-separation phases of violent relationships. Long-term impacts of violent relationships are often seen in research and practice as ‘posttraumatic,’ but our analysis demonstrates the importance of acknowledging the ongoing and persistent nature of the trauma arising from violent relationships. For our participants, violence did not stop once a relationship ended: they lived in a context of real, continuing threats of harm. Perpetrators of IPV used several mechanisms to continue post-separation abuse, including contact with children, stalking, “gaslighting,” and threats of lethal and nonlethal violence.

Our findings align with key concepts in the extant literature on CTS and demonstrate the utility of the concept for developing interventions and support services for IPV survivors. To recap: CTS (1) Is not located within individuals, but within contexts of “ongoing emergency” (Summerfield, 1999, p. 1459); (2) Is situated in an *ongoing temporal dimension*; (3) Involves a realistic appraisal of future danger; and (4) Exists in a context characterized by the absence of protections (Eagle & Kaminer, 2013). As we have shown, our participants’ accounts could be mapped on to these concepts, and this has important implications for reducing the physical, social, and psychological harms associated with IPV. In particular, the pervasive sense of threat can continue long after the end of a relationship, and so appropriate support for women to escape the immediate context is crucial. Legal sanctions against IPV perpetrators may be required, but these should be seen as part of a wider approach to IPV reduction. In fact, as our findings showed, while escaping the context and robust legal protections are necessary for survivors of IPV, they may not be sufficient: perpetrators continued to enact violence toward their partners even from prison in one case, and the continuous trauma often stretches back into childhood. Additionally, while our participants’ experiences showed that current protections from danger are inadequate, they are not entirely absent. With suitable intervention and legislation, women could better be supported to escape violence and process their trauma.

CTS was developed to address contexts of political violence and war (Straker & the Sanctuaries Counseling Team, 1987). Although the concept has not previously been applied to understanding the experiences of women post-separation from violent partners, the parallels are striking. In both cases, participants were often living in precarious settings, moving from one refuge to another; in both cases, participants were exposed to chronic violence and threats to life, and in both cases, perpetrators of violence are in a position of power relative to the victim. Finally, in both cases, the threat of violence was not situated in the past but was ongoing, exposing participants to chronic uncertainty and distress. At the same time, to understand IPV as a CTS phenomenon, we acknowledge the differences between experiences of IPV and of state violence. Perhaps most importantly, whereas CTS in situations of war or state repression emerges as a collective experience of violence against groups of people, CTS in the context of IPV is targeted against a particular individual. It is likely this difference will affect how continuous trauma is experienced, and for this reason different concepts of trauma have been developed in these situations—for example, historical trauma in the collective context (Gone, 2009), and developmental trauma in the individual context (van der Kolk, 2007). One of the key features of CTS is the notion that the perpetrator of violence is “faceless and unpredictable” (Eagle & Kaminer, 2013, p. 88). In IPV, although the perpetrator is similarly unpredictable (sometimes appearing remorseful and willing to change), the intimacy between the partners in the relationship is likely to alter the meaning and impact of trauma.

Our findings point to the need for a number of policy and practice changes to support and empower abused women. One key advantage of using the CTS concept to understand post-separation experiences is that it moves the focus away from an individualized understanding of IPV trauma toward one that situates the trauma in a wider context. With such a focus, psychological, social, and legal services would be encouraged to understand the complexity of post-separation trauma, and to design interventions that address the issues within the survivor’s life that constrain free choices within relationships—particularly the ongoing risk of harm from perpetrators. The choice of labels for describing post-separation also has implications for preventing further pathologization of IPV survivors’ choices and perspectives. In a different context—that of critical disability studies—understanding how “deficits” are rooted in the social environment has played a powerful role in reducing disability stigma, and on shifting the focus of intervention away from disabled bodies to disabling environments (e.g., Goodley, 2013). The social vocabulary implied in CTS has a use here too. For example, psychotherapy practice can benefit from addressing relational and social issues among clients, while promoting the individual-level strengths we saw among our participants (Bogat et al., 2013). CTS additionally shifts the focus away from the “problems” existing within survivors, and toward perpetrators’ behavior.

It should be recognized that women reporting IPV to the police have done so in a context of profound difficulties, and so their accounts by default should be taken seriously. However, it is also critical to realize that, in contacting the police, criminal charges against perpetrators may not be the primary aim of survivors: the main goal is ultimately to end the relationship safely (Hoyle & Sanders, 2017). In this respect, we suggest that effective policing for IPV requires working across professional boundaries, with social work professionals and third-sector organizations being linked up to find ways to ensure perpetrators face consequences for their actions, and to devote efforts to preventing IPV. IPV survivors are at high risk of violence after leaving a relationship (Brownridge, 2006), and so safety planning across sectors is vital. It is also crucial that domestic violence agencies receive the required funding to carry out their work assisting women on their journeys out of IPV—currently, services continue to operate in a context of an ongoing local authority spending freeze (IFS, 2020).

## Limitations

Despite efforts to recruit a broad sample of women, we spoke with few women who were not in touch with services and were unable to capture the experiences of those who may be especially isolated and marginalized. This is reflected in the lack of diversity in the sample; for example, in the small number of participants from black and minority ethnic groups, the lack of consideration of IPV within lesbian relationships and the failure to include disabled women. Consequently, we were not able to explore IPV within the context of racism, homophobia, and disablism and are aware of the need for further research in these areas. Also, given the recognition of children as victims of IPV, it is important that the relevance of CTS for understanding children’s experiences of IPV is a crucial topic for future research. However, despite these limitations, the inclusion of a spread of ages, parenting, and socioeconomic status, marginalized voices and, diversity in the types and duration of abuse experiences, has provided useful insights on post-separation abuse as CTS.

## Implications for Research and Practice

This study showed that post-separation abuse is not simply a continuation of the abuse that women experienced within violent relationships: separation may itself be a catalyst for new forms of control and abuse. For most of the women in this study, leaving a violent relationship was often a protracted process involving several attempts; this was primarily because of women’s fears of retributive violence. In the absence of effective protections, the risks were often perceived as being simply too great. Although post-separation abuse does not necessarily reflect the simple extension of pre-separation abuse, neither does it indicate two distinct abuse experiences (i.e., before and after); it is the imposition of new forms of controlling and threatening behaviors by ex-partners when one has reached a place and position of assumed safety that is concerning. IPV survivors experienced traumatic stress within abusive relationships, they faced traumatic stress when planning how and when to leave, and they experienced CTS after they had left because of ongoing abuse.

There is extensive research on IPV-related trauma, but the concept of CTS has yet to be applied. In assessing the impact of CTS, women may need different forms of supportive interventions from those traditionally envisaged. Using the CTS construct enables professionals to facilitate woman-centered appraisals of future threat and to develop strategies for its regulation and management. So, rather than focusing on cognitive intrusions from past abuse, the task “is to prepare for future traumatization and to develop the ability to discriminate between stimuli that might pose a real, immediate, or substantial threat from other everyday stimuli” (Eagle & Kaminer, 2013, p. 91). Actively enabling women to develop their intuitive skills and to work together with service providers to plan how to mitigate future abuse is an emancipatory approach which places women’s knowledge and experience at the core of service delivery. Additionally, effective constraints against perpetrators, and ways of holding them to account for past and continuing CTS, are critical. This requires that therapeutic services be provided as soon as possible, and secondly, that when women state that it is not safe for them to remain where they are for fear of post-separation abuse or potential death, the police and statutory protection services must engage with these fears and not only focus on “reality checking” that often result from women’s pleas for help.
